# VEGF-A promotes the motility of human melanoma cells through the VEGFR1–PI3K/Akt signaling pathway

**DOI:** 10.1007/s11626-022-00717-3

**Published:** 2022-08-23

**Authors:** Koichi Koizumi, Tomoaki Shintani, Yasutaka Hayashido, Atsuko Hamada, Mirai Higaki, Yukio Yoshioka, Akihiko Sakamoto, Souichi Yanamoto, Tetsuji Okamoto

**Affiliations:** 1 Department of Oral Oncology, Graduate School of Biomedical and Health Sciences, Hiroshima University, Hiroshima, 734-8553 Japan; 2 Center of Oral Clinical Examination, Hiroshima University Hospital, Hiroshima, 734-8551 Japan; 3 Oral Maxillofacial Surgery, Hiroshima University Hospital, 1-2-3 Kasumi, Minami-ku, Hiroshima, 734-8551 Japan; 4 Oral Maxillofacial Surgery, Hiroshima University Hospital, Hiroshima, 734-8551 Japan; 5 School of Medical Sciences, University of East Asia, Shimonoseki, 751-8503 Japan

**Keywords:** Melanoma, Cell motility, VEGF-A, VEGFR1, PI3K/Akt signaling pathway

## Abstract

Vascular endothelial growth factor A (VEGF-A) and its receptors (VEGFR1 and R2) play important roles in the progression of malignant melanoma through tumor angiogenesis. However, it is not clear whether the VEGF-A/VEGFR1 signaling pathway is involved in the proliferation and migration of melanoma cells. Thus, the effect of VEGF-A on cell migration was investigated in human melanoma cell lines. Of several splicing variants of VEGF-A, VEGF_165_ is the most abundant and responsible for VEGF-A biological potency. VEGF_165_ facilitated the migration of melanoma cells in both a chemotactic and chemokinetic manner, but cell proliferation was not affected by VEGF_165_. VEGF_165_ also induced the phosphorylation of Akt. In addition, VEGF_165_-induced cell migration was inhibited significantly by VEGFR1/2 or a VEGFR1-neutralizing antibody. Furthermore, the downregulation of VEGFR1 via the transfection of VEGFR1-targeting antisense oligonucleotides suppressed VEGF_165_-induced cell migration. Moreover, wortmannin, an inhibitor of phosphatidylinositol-3 kinase (PI3K) in the PI3K/Akt pathway, suppressed VEGF_165_-induced Akt phosphorylation and VEGF_165_-induced cell migration. These findings suggest that the motility of melanoma cells is regulated by signals mediated through the PI3K/Akt kinase pathway with the activation of VEGFR1 tyrosine kinase by VEGF_165_. Thus, the downregulation of signaling via VEGF-A/VEGFR1 might be an effective therapeutic approach that could prevent the progression of malignant melanoma.

**Supplementary Information:**

The online version contains supplementary material available at 10.1007/s11626-022-00717-3.

## Introduction

One characteristic property of malignant tumors is their ability to invade the surrounding tissues and form metastatic foci in distant organs. Metastasis involves a series of steps, including the detachment of cancer cells from the primary lesion, migration into connective tissues, intravasation into the circulation, and implantation into distant organs (Bravo-Cordero *et al*. 2012; Clark and Vignjevic 2015).

Tumor cells are known to produce growth factors and cytokines, such as vascular endothelial growth factor (VEGF), transforming growth factors, and basic fibroblast growth factors, which have various biological activities in tumor cells and stroma cells, including endothelial cells and fibroblasts (Hayashido *et al*. 1998; Guo *et al*. 2021; Motwani and Eccles 2021). VEGF is a potent angiogenic factor that binds to two tyrosine kinase-type receptors, VEGF receptor-1 (VEGFR1)/fms-like tyrosine kinase (Flt-1) and VEGFR2/kinase insert domain receptor (KDR)/fetal liver kinase 1, which are specifically and highly expressed in vascular endothelial cells. The interaction of VEGF and VEGFRs has a stimulatory effect on the proliferation and migration of vascular endothelial cells (Vaisman *et al*. 1990; Myoken *et al*. 1991). Importantly, VEGF is known to be upregulated in several tumors and to contribute to tumor angiogenesis.

The VEGF family consists of VEGF-A–E and placental growth factor (PlGF). VEGF-A plays a central role in tumor angiogenesis in relation to blood vessel sprouting, repair, and regeneration (Dvorak 2021). VEGF-A consists of several splice variants with different numbers of amino acids, such as VEGF_121_, VEGF_145_, VEGF_165_, and VEGF_189_. VEGF_165_ is the most abundant and responsible for VEGF-A biological potency (Dvorak 2021). Although VEGF-A binds to both VEGFR1 and VEGFR2, VEGF-B and PlGF bind only to VEGFR1. The affinity of VEGF-A to VEGFR1 is about tenfold higher than its affinity to VEGFR2, whereas the tyrosine kinase activity of VEGFR1 is about tenfold lower than that of VEGFR2 (Shibuya 2006, 2011; Apte *et al*. 2019). VEGFR1 contributes to pathological angiogenesis in tumors, rheumatoid arthritis, and cerebral ischemia, and VEGFR2 is the regulator of both physiological and pathological angiogenesis (Dvorak 2021). Although PlGF is not involved in physiological angiogenesis, it participates in pathological angiogenesis in cancer tissues via VEGFR1 (Dewerchin and Carmeliet 2012).

In general, tumor cells have the ability to produce VEGFs, whereas their expression of VEGFRs is strongly suppressed. Previous studies have shown that VEGFRs are expressed in many types of cancers, including melanoma, pancreatic, lung, and ovarian cancers, suggesting that VEGFs might regulate tumor progression through not only paracrine mechanisms but also autocrine mechanisms (Gitay-Goren *et al*. 1993; Frank *et al*. 2011; Shibuya 2011; Borsotti *et al*. 2015).

Melanoma is a malignant tumor derived from melanocytes in the skin and mucous membrane (Iversen and Robins 1980; Yde *et al*. 2018; Ahmed *et al*. 2020). Melanoma frequently metastasizes due to its ability to migrate effectively and form a vascular network in tumor tissues (Streit and Detmar 2003; Pasquali *et al*. 2018). Moreover, melanoma is known to express high levels of PlGF and VEGF-A. In vivo studies have shown that when melanoma cells are inoculated into transgenic mice that overexpress PlGF, tumor growth is increased significantly and metastatic potential is relatively higher than that in control mice inoculated with melanoma cells (Lacal *et al*. 2000; Graziani *et al*. 2016; Lacal and Graziani 2018). Furthermore, VEGFR1-expressing melanoma cells have been shown to be more invasive compared with melanoma cells that do not express VEGFR1, and the blockade of VEGFR1 using a specific monoclonal antibody reduces VEGF‐A- and PlGF-inducible extracellular matrix invasion (Hennequin *et al*. 1999). These results suggest that a signal mediated via VEGFR1 might regulate the invasion of melanoma cells. However, the mechanism underlying the tumor-produced VEGF-regulated invasion and metastasis of melanoma remains unclear. Thus, in the present study, we examined the expression of VEGF-A and VEGFR1 in human melanoma cells and investigated the effects of VEGF_165_/VEGFR on the migration and proliferation of human melanoma cells as well as the VEGF_165_/VEGFR-related signaling pathway.

## Materials and methods

### Chemicals and antibodies

Insulin, transferrin, 2-aminoethanol, sodium selenite, 2-mercaptoethanol, oleic acid conjugated with fatty acid–free bovine serum albumin (BSA), and PlGF were purchased from Sigma-Aldrich (St. Louis, MO). Recombinant Human VEGF_165_ and Human VEGF Quantikine ELISA Kits were obtained from R&D Systems Inc. (Minneapolis, MN). Type I collagen solution (Native Collagen Acidic Solution, IAC-50) was purchased from Koken (Tokyo, Japan). The VEGFR1/2 tyrosine kinase activity inhibitor [CB676475, (4-[(4′-chloro-2′-fluoro) phenylamino]-6,7-dimethoxyquinazoline)] was purchased from Calbiochem (San Diego, CA), and the VEGFR2 kinase inhibitor II [(Z)-5-bromo-3-[(4,5,6,7-tetrahydro-1H-indol-2-yl) methylene]-1,3-dihydroindol-2-one] was purchased from Merck Biosciences (Nottingham, UK). Wortmannin, a kinase inhibitor of phosphatidylinositol-3 kinase (PI3K), was obtained from Sigma-Aldrich.

Rabbit polyclonal anti-phospho-VEGFR1 antibody (Y1059; CSB-PA000747) and rabbit polyclonal anti-phospho-VEGFR2 antibody (Y1048; CSB-PA009634) were purchased from Cusabio Technology (Houston, TX). Rabbit polyclonal anti-VEGFR1 antibody (A1277) and rabbit polyclonal anti-VEGFR2 antibody (A5609) were purchased from ABclonal (Boston, MA). Rabbit monoclonal anti-phosphorylated Akt antibody (Ser473; #4060), rabbit monoclonal anti-phosphorylated extracellular signal-regulated kinase-1/2 (Erk1/2) (Thr202/Tyr204; #4370), rabbit monoclonal anti-Akt antibody (#4685), anti-Erk1/2 antibody (#4695), rabbit monoclonal anti-β-Actin (#4970), and horseradish peroxidase (HRP)–conjugated anti-rabbit IgG antibody (#7074) were purchased from Cell Signaling Technology (Danvers, MA). Anti-VEGFR1 blocking monoclonal antibody (KM1750) was kindly provided by Dr. Shibuya (Jobu University, Isesaki, Japan) and Dr. Shitara (Kyowa Hakko Kirin Co., Ltd, Tokyo, Japan).

### Cells and culture

The human melanoma cell lines SK-MEL-28 (RRID:CVCL_0526) (Shiku *et al*. 1976), HMV-II (RRID:CVCL_1282) (Kasuga *et al*. 1976), G361 (RRID:CVCL_1220) (Peebles *et al*. 1978), and C32TG (RRID:CVCL_2324) (Jia *et al*. 1997) were provided by RIKEN BRC (Tsukuba, Japan) and used in this study. Malignant melanoma (MM) cells established in our laboratory from a patient with malignant melanoma of the gingiva were also used (Okamoto *et al*. 1996). These cell lines are free from mycoplasma contamination using e-Myco™ plus Mycoplasma PCR Detection Kit (iNtRON, Seongnam-Si, South Korea) and have been authenticated using short tandem repeat (STR) profiling (BEX Co., Ltd., Tokyo, Japan) within the last 3 mo (Supplementary Figure S1 and Table S1). The STR profiles of these cell lines, except for MM cell line, matched with the publicly available reference profiles (ICLAC Databases. 2021). As MM cell line has not yet deposited to the cell bank, the STR profile of MM cell line did not match with any other STR data in the databases.

All cells were grown in DF medium (1:1 mixture (by volume) of Dulbecco’s modified Eagle medium (DMEM) and Ham F-12 medium) supplemented with 5% fetal bovine serum in a humidified 95% air/5% CO_2_ atmosphere at 37 °C in a CO_2_ incubator (Thermo Fisher Scientific, Waltham, MA). Cell proliferation was estimated as follows. The wells of 24-well tissue culture plates were coated with 100 µg/mL of type I collagen, and cells (5 × 10^3^) suspended in DF 6F serum–free medium supplemented with 10 µg/mL of insulin, 5 µg/mL of transferrin, 10 µM of 2-aminoethanol, 10 nM of sodium selenite, 10 µM of 2-mercaptoethanol, and 9.4 µg/mL of oleic acid conjugated with fatty acid–free BSA were seeded in each well of the culture plates (Sato *et al*. 1987). After 24 h, various concentrations of VEGF_165_ were added, and the cells were cultured in 5% CO_2_ for 5 d at 37 °C. Subsequently, the number of cells was counted using a Coulter counter (Beckman Coulter, Tokyo, Japan), and the measurements were collected in triplicate. All reagents used in the cell culture were free from mycoplasma and viral pathogens.

### RNA extraction and RT-PCR for VEGF-A and VEGFR mRNAs

Total RNA was isolated from the cells using TRIzol reagent (Thermo Fisher Scientific) following the manufacturer’s protocol, and RNA quality was determined according to the following criteria: RNA concentration > 0.5 µg/µL; OD 260/280 = 1.8–2.0. Reverse transcription was performed using the Super Script First-strand Synthesis System (Life Technologies, Carlsbad, CA). PCR was performed for VEGF-A and VEGFRs with glyceraldehyde-3-phosphate dehydrogenase (GAPDH) used as the internal control. Following an initial incubation at 94 °C, each PCR cycle consisted of incubation for 30 s at 94 °C, 30 s at 55 °C, and 1 min at 72 °C. After the final cycle, the samples were incubated for a further 7 min at 72 °C and then kept at 4 °C before analysis via agarose gel electrophoresis. The following primers were used: VEGF-A forward primer, 5′-CTTGCCTTGCTGCTCTACC-3′; VEGF-A reverse primer, 5′-CACACAGGATGGCTTGAAG-3′; VEGFR1 forward primer, 5′-CATGAGGATGAGAGCTCCTGAG-3′; VEGFR1 reverse primer, 5′-AGGCCAACAGAGTGCTGCTGTC-3′; VEGFR2 forward primer, 5′-CCTGTCCACTTACCTGAGGAG-3′; VEGFR2 reverse primer, 5′-CTGGCTACTGGTGATGCTGTC-3′; GAPDH forward primer, 5′-GCTCTCTGCTCCTCCTGTTC-3′; and GAPDH reverse primer, 5′-ACGACCAAATCCGTTGACTC-3′.

### Immunoblot analysis

Cells were lysed using cell lysis buffer (50-mM Tris HCl (pH 7.4), 150-mM NaCl, 1-mM EDTA, 1% Triton X-100, 0.1% sodium dodecyl sulfate (SDS), and 0.5% sodium deoxycholate) supplemented with 1% protease inhibitor cocktail (Sigma-Aldrich). The lysates were centrifuged at 15,000 × *g* and 4 °C for 15 min, and the supernatants were collected. Samples containing 20 μg of total protein were electrophoresed on 10% SDS–polyacrylamide gel under reducing conditions and transferred to polyvinylidene difluoride (PVDF) membrane filters (Bio-Rad Laboratories, Hercules, CA). The filters were blocked using TBS-T (20-mM Tris HCl (pH 7.5), 137-mM NaCl, and 0.1% Tween 20) containing 5% skim milk for 1 h at room temperature, after which they were incubated with primary antibodies and then with HRP-conjugated secondary antibody. Rabbit monoclonal anti-β-actin was used as a loading control antibody. Protein bands were visualized using enhanced chemiluminescence detection (Clarity ECL Substrate; Bio-Rad Laboratories).

### ELISA for soluble VEGF_165_

To obtain conditioned media, 80% confluent melanoma cells in 6-well plates were washed twice with DF and incubated with 2 mL of DF for 24 h. The conditioned media were then centrifuged at 10,000 × *g* and 4 °C for 30 min to remove cells and debris. The amount of soluble VEGF_165_ in the conditioned media was measured using a Human VEGF Quantikine ELISA Kit according to the manufacturer’s instructions. The levels of VEGF_165_ detected were corrected according to the number of cells.

### Cell motility assay

Cell motility was analyzed using a modified Boyden chamber assay with Transwell inserts (6.5 mm in diameter) containing 8-μm pores (Corning Costar, Cambridge, MA) as described previously (Chen 2005; Hayashido *et al*. 2007). The filters were coated with 100 μg/mL of type-1 collagen to enhance cell attachment. Melanoma cells (1 × 10^5^) resuspended in DF medium containing 0.1% BSA were added to the upper compartment of each Transwell insert, and VEGF_165_ or PlGF (Sigma-Aldrich) was added to the upper or lower compartment. After incubation for 24 h at 37 °C, the Transwell inserts were fixed with methanol and stained with Diff-Quik (Dade Behring AG, Dudingen, Switzerland). The cells on the upper surface of the filter were wiped with a cotton swab, and the number of cells on the lower surface of the filter was counted under a low-power field (× 50) using light microscopy. Five fields were counted in each of the three different experiments, and the results were expressed as the mean number of migrating cells/mm^2^ ± the standard deviation (SD). To assess the chemotactic or chemokinetic response of VEGF_165_, checkerboard analysis was performed by adding various concentrations of VEGF_165_ to both the lower and upper Transwell chambers.

### Phosphorylation assay

Melanoma cells were cultured on 6-well plates until near confluence and starved with serum-free DF overnight. The cells were then incubated with 5 ng/mL of recombinant human VEGF_165_ for the indicated periods, washed with ice-cold phosphate-buffered saline containing 1 mM of sodium vanadate, and lysed with cell lysis buffer supplemented with protease inhibitor cocktail and 1 mM of sodium vanadate. The samples were separated on 10% SDS–polyacrylamide gels under reducing conditions and transferred onto PVDF membrane filters. The phosphorylation of VEGFR1 and VEGFR2 was examined using immunoblotting with rabbit polyclonal anti-phospho-VEGFR1 antibody and rabbit polyclonal anti-phospho-VEGFR2 antibody, respectively. The phosphorylation of ERK1/2 and Akt was assessed using rabbit anti-phospho-ERK1/2 monoclonal antibody and rabbit anti-phospho-Akt monoclonal antibody, respectively. Total VEGFR1, VEGFR2, Akt, and MEK1/2 were detected using rabbit anti-VEGFR1 antibody, rabbit polyclonal anti-VEGFR2 antibody, rabbit anti-ERK1/2 monoclonal antibody, and rabbit anti-Akt monoclonal antibody, respectively. After incubation with the primary antibodies, the membranes were incubated with HRP-conjugated secondary antibody, and protein bands were detected using an enhanced chemiluminescence reagent.

### Antisense oligonucleotides (ASOs) and transfections

To downregulate VEGFR1 or VEGFR2, morpholino antisense ASOs specific for VEGFR1 or VEGFR2 (GeneTools, Philomath, OR) were used. The sequences of the ASOs were as follows: VEGFR1, 5′-AAGCCAGGGCCGAGCCGCACATAAT-3′; VEGFR2, 5′-GCAGCACCTTGCTCTGCATCCTGCA-3′. A standard control morpholino oligonucleotide (5′-CCTCTTACCTCAGTTACAATTTATA-3′) was used as a negative control. Delivery of the oligonucleotides into the cells was performed according to the GeneTools protocol. Briefly, 80–100% confluent SK-MEL-28 cells were treated with 10 μM of the morpholino ASOs or the standard control oligonucleotide and 6 μM of Endo-Porter reagent (GeneTools). After 24 h, the cells were used in the subsequent experiments.

### Statistical analysis

Statistical analysis was performed using BellCurve for Excel (Social Survey Research Information Co., Ltd., Tokyo, Japan). All data are presented as the means ± SD of at least three independent experiments. Student’s *t-*test was used to compare the differences between groups, which were considered significant at *p* < 0.05.

## Results

### Expression of VEGF-A, VEGFR1, and VEGFR2 in melanoma cells

**Figure 1.**
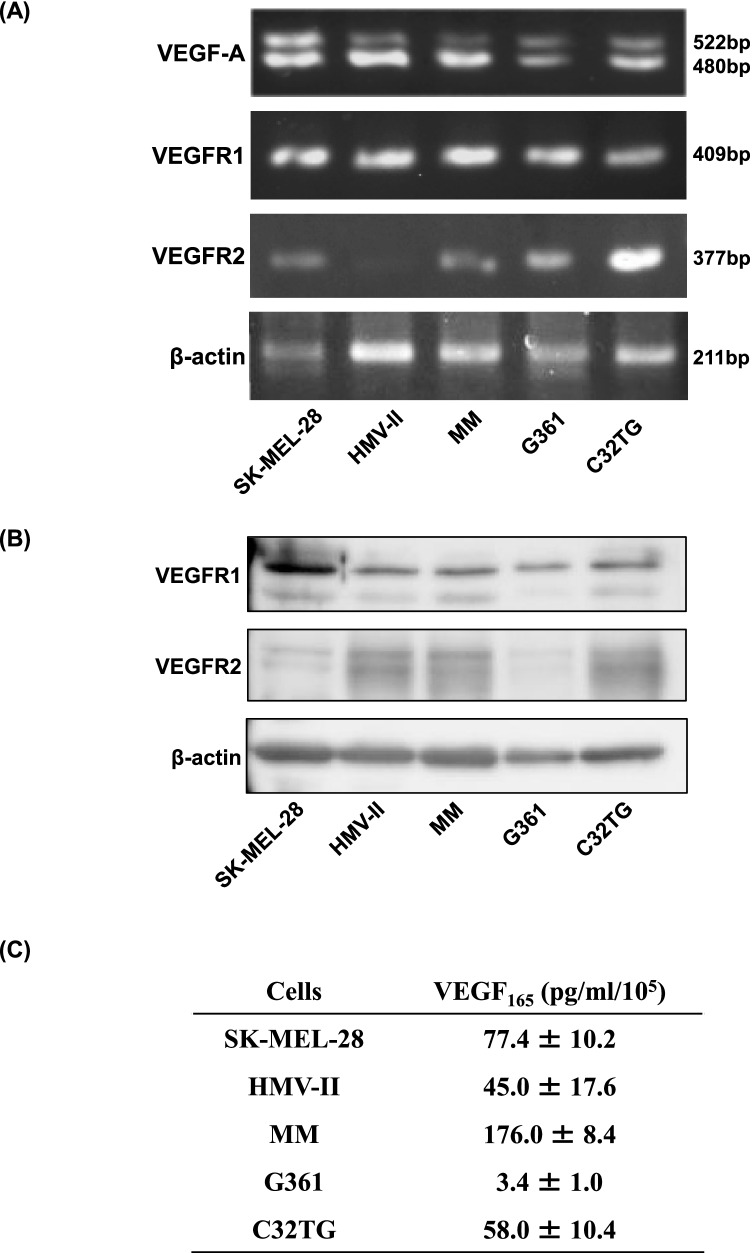
Expression of vascular endothelial growth factor (VEGF)-A, VEGFR1, and VEGFR2 in human melanoma cells. Total RNA was extracted from melanoma cells, and the expression of VEGF-A, VEGFR1, and VEGFR2 mRNAs was analyzed using RT-PCR. All cells expressed the mRNAs of VEGF_165_ and VEGF_189_ (*A*), and the expression of VEGFR1 and VEGFR2 mRNAs (*B*) was also observed. Melanoma cells (80% confluent) were cultured in serum-free medium for 24 h, and VEGF_165_ protein in the culture supernatants was quantified using ELISA (*C*).

The mRNA expression of VEGF-A, VEFGR1, and VEGFR2 in melanoma cells was examined using RT-PCR. The PCR products of VEGF_121_, VEGF_165_, and VEGF_189_, which are splicing variants of VEGF-A, were detected in all cell lines (Fig. 1*A*), as was the expression of VEGFR1/Flt-1 and VEGFR2/KDR mRNAs (Fig. 1*B*). VEFGR1 and VEGFR2 protein expression was examined using immunoblotting. VEGFR1 protein was expressed in all melanoma cells, whereas VEGFR2 protein was not detected via immunoblotting. To investigate VEGF_165_ secretion by melanoma cells, the amount of VEGF_165_ in the conditioned media was assayed using an ELISA. The concentrations of VEGF_165_ in the conditioned media were as follows: 77.4 ± 10.2 pg/mL/10^5^ cells in SK-MEL-28 cells; 45.0 ± 17.6 pg/mL/10^5^ cells in HMV-II cells; 176.0 ± 8.4 pg/mL/10^5^ cells in MM cells; 3.4 ± 1.0 pg/mL/10^5^ cells in G361 cells; and 58.0 ± 10.4 pg/mL/10^5^ cells in C32TG cells (Fig. 1*C*).

### Effect of VEGF_165_ on the proliferation of melanoma cells

**Figure 2.**
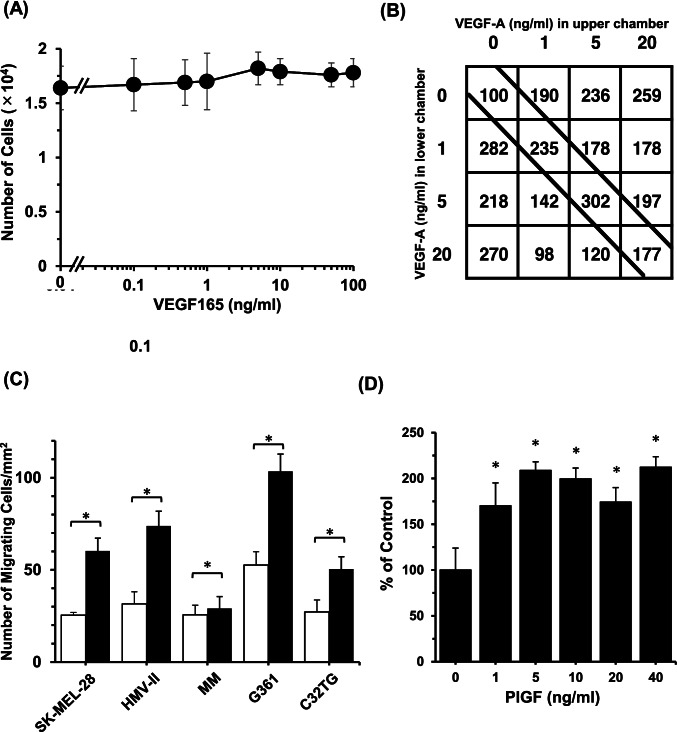
Effect of VEGF_165_ on the proliferation and motility of melanoma cells. SK-MEL-28 cells (5 × 10^3^) suspended in DF 6F serum–free medium containing 10 µg/mL of insulin, 5 µg/mL of transferrin, 10 µM of 2-aminoethanol, 10 nM of sodium selenite, 10 µM of 2-mercaptoethanol, and 9.4 µg/mL of oleic acid conjugated with fatty acid–free bovine serum albumin (BSA) were seeded in each well of a 24-well tissue culture plate coated with type-I collagen. After 24 h, the indicated concentrations of VEGF_165_ were added. The number of cells was measured after cultivation for 5 d (*A*). The effect of VEGF_165_ on the migration of SK-MEL-28 cells was investigated using a modified Boyden chamber method. SK-MEL-28 cells (1 × 10^5^) suspended in DF medium containing 0.1% BSA and the indicated concentrations of VEGF_165_ were added to the upper and lower chambers. After incubation for 24 h at 37 °C, the number of cells that had migrated to the lower surface of the filter was counted a percentage of the untreated control. Cell motility was estimated using checkerboard analysis (*B*). Melanoma cells (1 × 10^5^) were added to the upper chambers and cultured with ( +) or without ( −) 5 ng/mL of VEGF_165_ in both the upper and lower chambers. After cultivation for 24 h, the number of cells that had migrated was counted (*C*). SK-MEL-28 cells (1 × 10^5^) were added to the upper chamber, and the indicated concentrations of PlGF were added to both the upper and lower chambers. After cultivation for 24 h, the number of cells that had migrated was counted (*D*). All experiments were performed in triplicate, and data are means ± SD. In *B* and *D*, the data represent percentages of the untreated control. *p < 0.05.

VEGF_165_ at concentrations of 0.1–100 ng/mL did not affect the proliferation of SK-MEL-28 cells (Fig. 2*A*) or the proliferation of the other melanoma cells (data not shown). By contrast, purified VEGF_165_ half-maximally stimulated human umbilical vein endothelial cell (HUVEC) proliferation at 41 pM (1.8 ng/mL) and maximally stimulated HUVEC growth at 200 pM (8.8 ng/mL) (Myoken *et al*. 1991).

### Effect of VEGF_165_ on the migration of melanoma cells

The effect of VEGF_165_ on the migration of melanoma cells was analyzed using a modified Boyden chamber method. Checkerboard analysis indicated that VEGF_165_ induced chemotactic and chemokinetic migration in SK-MEL-28 cells, and 5 ng/mL of VEGF_165_ added to both the upper and lower chambers led to the highest enhancement of migration (Fig. 2*B*). In all melanoma cells, 5 ng/mL of VEGF_165_ added to the upper and lower chambers enhanced migration significantly (*p* < 0.05) compared with that in the controls (Fig. 2*C*).

### Effect of PIGF on the proliferation and migration of melanoma cells

To clarify whether the VEGF_165_-induced migration of melanoma cells is regulated via VEGFR1 or VEGFR2, the effect of PlGF on the migration of melanoma cells was investigated. PlGF increased cell migration significantly (*p* < 0.05) in a dose-dependent manner (Fig. 2*D*) but did not affect cell proliferation (data not shown). These results suggested that VEGF_165_-induced migration of melanoma cells was mediated only by VEGFR1.

### Participation of VEGFRs in the migration of melanoma cells

**Figure 3.**
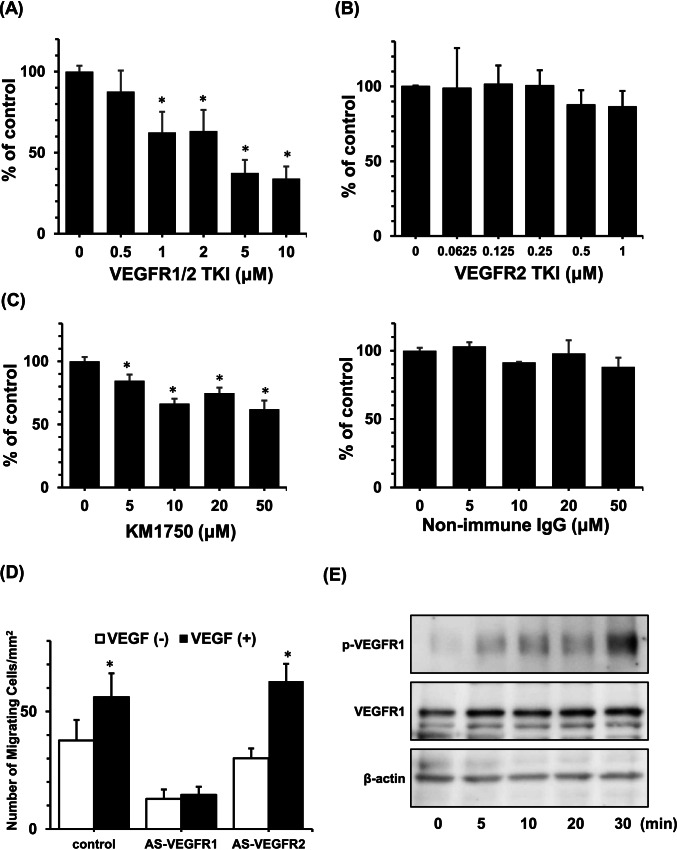
Effects of VEGFR tyrosine kinase inhibitors (TKIs), VEGFR1-neutralizing antibody, and VEGFR antisense oligonucleotides (ASOs) on the VEGF_165_-mediated motility of melanoma cells. SK-MEL-28 cells treated with 4-[(4′-chloro-2′-fluoro) phenylamino]-6,7-dimethoxyquiazorine, a VEGFR1/2 TKI, for 1 h were suspended in DF medium containing 0.1% BSA and then added to the upper compartments of Transwell chambers. After incubation with 5 ng/mL of VEGF_165_ in both the upper and lower compartment for 24 h at 37 °C, the number of cells that migrated to the lower surface of the filter was counted. The VEGFR1/2 TKI suppressed VEGF_165_-induced cell migration in a dose-dependent manner (*A*). After the treatment of SK-MEL-28 cells with (Z)-5-bromo-3-[(4,5,6,7-tetrahydro-1H-indol-2-yl) methylene]-1,3-dihydroindol-2-one, a VEGFR2 TKI, for 1 h, the number of cells that migrated when incubated with 5 ng/mL of VEGF_165_ in both the upper and lower chambers for 24 h was determined. The VEGFR2 TKI did not affect VEGF_165_-induced cell migration (*B*). After treatment of SK-MEL-28 cells with KM1750, a VEGFR1-neutralizing antibody, or nonimmune IgG for 1 h, the number of cells that migrated in the presence of 5 ng/mL of VEGF_165_ was investigated using a modified Boyden chamber assay. KM1750 suppressed VEGF_165_-induced cell migration in a dose-dependent manner (*C*). The motility of SK-MEL-28 cells transfected with VEGFR1 or VEGFR2 ASOs was investigated using a modified Boyden chamber assay. The motility of SK-MEL-28 cells transfected with control oligonucleotide or VEGFR2 ASO was enhanced in terms of migration induced by 5 ng/mL of VEGF_165_. The transfection of VEGFR2 ASO significantly reduced the migration of SK-MEL-28 cells, and the addition of VEGF_165_ did not enhance cell motility (*D*). SK-MEL-28 cells were cultured in the presence of VEGF_165_ for the indicated periods, and the expression of phosphorylated VEGFR1 (p-VEGFR1) was analyzed using immunoblotting. VEGFR1 phosphorylation was observed 5 min after treatment with VEGF_165_ (*E*). In *A*–*D*, data are represented as percentages of the untreated control and are the means ± SD of three replicates. *p < 0.05.

SK-MEL-28 cells treated with CB676475 VEGFR1/2 tyrosine kinase inhibitor (TKI), VEGFR2 kinase inhibitor II, or anti-VEGFR1 blocking monoclonal antibody for 1 h were then suspended in DMEM containing 0.1% BSA and added to an upper Transwell compartment. After incubation in the presence of 5 ng/mL of VEGF_165_ for 24 h, the number of cells that migrated to the lower surface of the filter was counted. Treatment with VEGFR1/2 TKI led to the suppression of VEGF_165_-induced cell motility in a dose-dependent manner (Fig. 3*A*). In contrast, VEGFR2 TKI did not alter VEGF_165_-induced cell motility (Fig. 3*B*). The treatment of SK-MEL-28 cells with KM1750, a neutralizing antibody for VEGFR1, also suppressed VEGF_165_-induced cell migration in a dose-dependent manner (Fig. 3*C*).

The suppression of VEGFR1 by a morpholino ASO targeting VEGFR1 led to a marked decrease in the migration of SK-MEL-28 cells, and the migration of SK-MEL-28 cells transfected with a VEGFR1-targeting morpholino ASO was not stimulated by VEGF_165_ (Fig. 3*D*). Conversely, the migration of SK-MEL-28 cells transfected with a VEGFR2-targeting morpholino ASO was enhanced by VEGF_165_. These experiments with inhibitors of VEGF receptor activities confirm the conclusion from the experiments with PIGF-treated melanomas that VEGF_165_ stimulated melanoma migration through VEGFR1. To assess VEGFR signaling in melanoma cells, the phosphorylation of VEGFRs was analyzed using western blot analysis following the addition of VEGF_165_. Treatment with VEGF_165_ led to phosphorylation of VEGFR-1 (Fig. 3*E*).

### Participation of VEGF_165_ in the ERK signaling pathway

**Figure 4.**
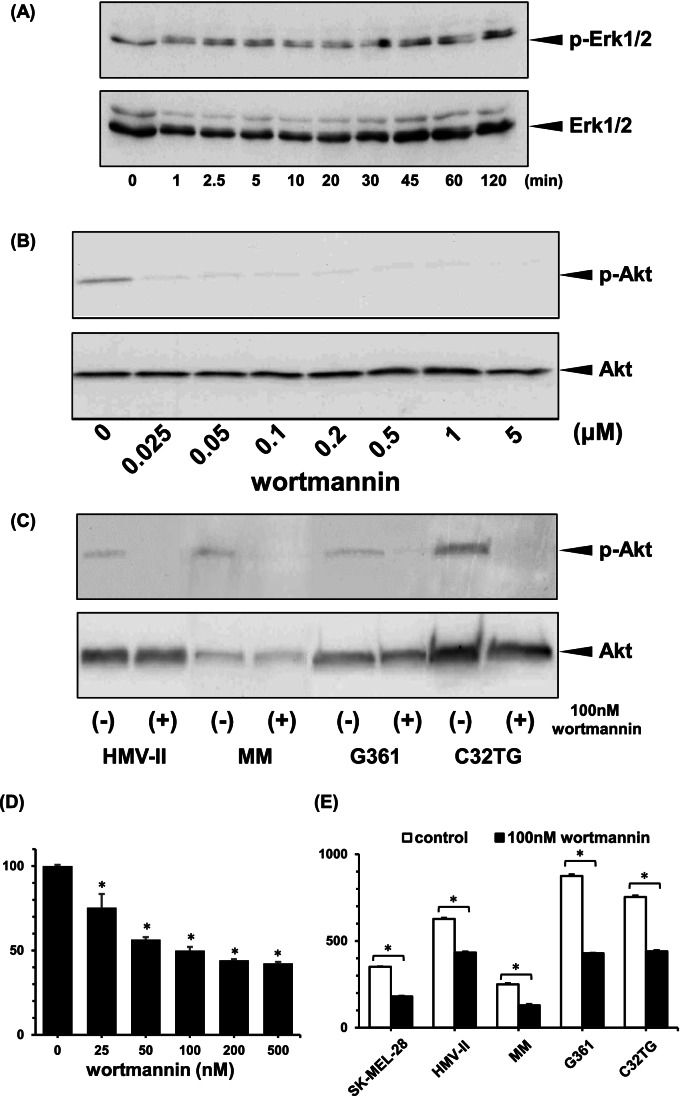
Effects of a PI3K inhibitor on the VEGF_165_-mediated motility of melanoma cells. SK-MEL-28 cells were cultured in the presence of VEGF_165_ for the indicated periods, and the expression of phosphorylated Erk (p-Erk) and phosphorylated Akt (p-Akt) was analyzed using immunoblotting. SK-MEL-28 cells expressed p-Erk constitutively, and VEGF_165_ did not alter Erk phosphorylation (*A*). After the treatment of SK-MEL-28 cells with the indicated concentration of wortmannin, a PI3K inhibitor, for 1 h, the cells were cultured with 5 ng/mL of VEGF_165_ for 1 h, and the phosphorylation of Akt was then examined using immunoblotting. Wortmannin suppressed the VEGF_165_-induced phosphorylation of Akt in a dose-dependent manner (*B*). After treatment with 100 nM of wortmannin for 1 h, HMV-II, MM, G361, and C32TG cells were cultured with 5 ng/mL of VEGF_165_ for 1 h, and the phosphorylation of Akt was then examined. VEGF_165_-induced phosphorylation of Akt was suppressed in all cells (*C*). After the treatment of SK-MEL-28 cells with the indicated concentrations of wortmannin for 1 h, the motility of SK-MEL-28 cells cultured with 5 ng/mL of VEGF_165_ for 24 h was analyzed using a modified Boyden chamber assay. Wortmannin suppressed the VEGF_165_-induced migration of SK-MEL-28 cells in a dose-dependent manner (*D*). After the treatment of melanoma cells with 100 nM of wortmannin for 1 h, the number of cells that migrated following incubation with 5 ng/mL of VEGF_165_ for 24 h was analyzed using a modified Boyden chamber assay. Wortmannin suppressed VEGF_165_-induced cell migration significantly (*E*). In *D* and *E*, data are represented as percentages of the untreated control. All data are the means ± SD of three replicates. *p < 0.05.

To examine the effect of VEGF_165_ on Erk phosphorylation in melanoma cells, the expression of phosphorylated Erk in SK-MEL-28 cells cultivated with VEGF_165_ was analyzed using immunoblotting. Phosphorylated Erk was expressed constitutively in SK-MEL-28 cells, and VEGF_165_ did not alter the expression of phosphorylated Erk (Fig. 4*A*).

### Participation of the PI3K/AKT signaling pathway in VEGF_165_-induced cell migration

The participation of PI3K in Akt phosphorylation was investigated using wortmannin, a PI3K inhibitor, which suppressed VEGF_165_-induced Akt phosphorylation in SK-MEL-28 cells in a dose-dependent manner (Fig. 4*B*). Similarly, wortmannin suppressed VEGF_165_-induced Akt phosphorylation in the other melanoma cells (Fig. 4*C*). Wortmannin also suppressed VEGF_165_-induced SK-MEL-28 cell migration in a dose-dependent manner (Fig. 4*D*), and it suppressed VEGF_165_-induced cell migration significantly (*p* < 0.05) in the other melanoma cells (Fig. 4*E*).

### Activation of the PI3K/AKT signaling pathway by VEGFR1

**Figure 5.**
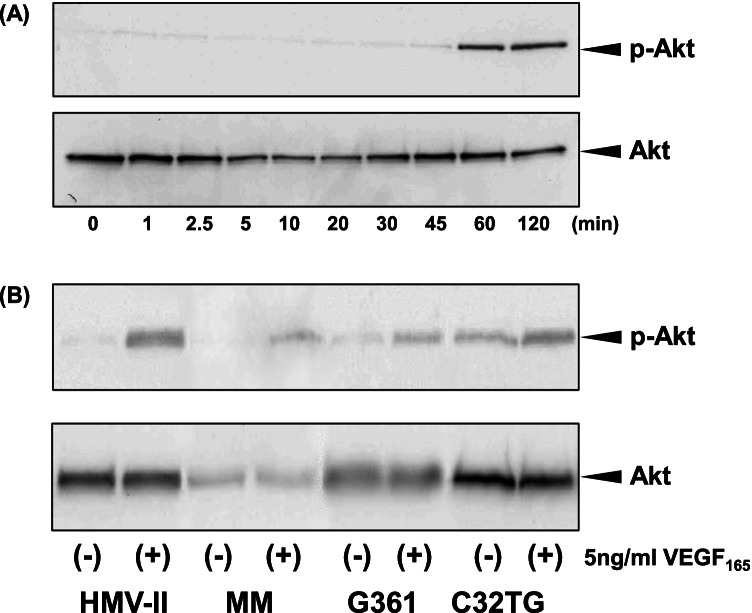
Effects of VEGF_165_ on mitogen-activated protein kinase and phosphatidylinositol-3 kinase (PI3K)/Akt activation in melanoma cells. SK-MEL-28 cells were cultured in the presence of VEGF_165_ for the indicated periods, and the expression of phosphorylated Akt (p-Akt) was analyzed using immunoblotting. Akt phosphorylation was observed 1 h after treatment with VEGF_165_ (*A*). Other melanoma cells were cultured for 1 h with or without 5 ng/mL of VEGF_165_, and Akt phosphorylation was then analyzed using immunoblotting. p-Akt was observed in all cells treated with VEGF_165_ (*B*).

**Figure 6.**
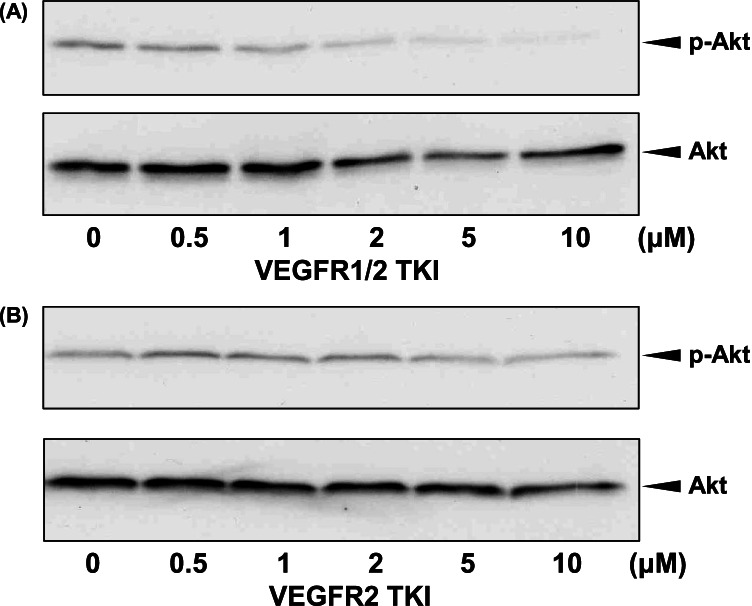
Effects of VEGFR TKIs on VEGF_165_-induced Akt phosphorylation. After the treatment of SK-MEL-28 cells with VEGFR1/2 TKI or VEGFR2 TKI for 1 h, the cells were cultured in the presence of 5 ng/mL of VEGF_165_ for 1 h, and the expression of p-Akt was then examined using immunoblotting. VEGFR1/2 TKI suppressed Akt phosphorylation by VEGF_165_ in a dose-dependent manner (*A*). VEGFR2 TKI did not alter Akt phosphorylation (*B*).

The effect of VEGF_165_ on the phosphorylation of Akt in SK-MEL-28 cells was analyzed using immunoblotting. Phosphorylated Akt was observed 1 h after the VEGF_165_ treatment was applied (Fig. 5*A*). Furthermore, the VEGF_165_ treatment led to phosphorylation of Akt in other melanoma cells (Fig. 5*B*). To determine whether VEGF_165_-induced Akt phosphorylation is regulated via VEGFR1 or VEGFR2, the effects of VEGFR1- or VEGFR2-targeting TKIs on VEGF_165_-induced Akt phosphorylation were investigated. VEGFR1/2 TKI suppressed the VEGF_165_-induced phosphorylation of Akt (Fig. 6*A*), whereas VEGFR2 TKI did not alter the expression of phosphorylated Akt (Fig. 6*B*). These results suggest that the PI3-kinase pathway in melanomas is activated by VEGFR1 but not VEGFR2.

## Discussion

The expression of VEGF-A in malignant tumors is closely related to tumor progression and prognosis (Aoyagi *et al*. 2010; Martins *et al*. 2013). The biological functions of VEGF-A are exerted through its binding to two tyrosine kinase receptors, VEGFR1 and VEGFR2, expressed in vascular endothelial cells. VEGF-A plays an important role in tumor angiogenesis by enhancing the proliferation and motility of endothelial cells (Motwani and Eccles 2021). Several studies have shown that various cancer cells produce VEGF-A and express its receptors, VEGFR1 and/or VEGFR2 (von Marschall *et al*. 2000; Carrillo de Santa Pau *et al*. 2009; Hlobilkova *et al*. 2009; Sopo *et al*. 2019). Thus, VEGF-A could promote tumor development and progression by regulating the proliferation and motility of tumor cells in an autocrine manner as well as angiogenesis induction in a paracrine manner.

In the present study, we first examined the expression of VEGF-A, VEGFR1, and VEGFR2 in melanoma cells as well as their participation in the motility and proliferation of these cells. All melanoma cells tested secreted VEGF_165_ into the culture media and expressed VEGFR1 and VEGFR2, suggesting that the VEGF_165_ produced by melanoma cells might regulate the proliferation and motility of these cells in an autocrine manner. In addition, radio-receptor assay using [^125^ I]-labeled VEGF_165_ confirmed that SK-MEL-28 cell line expressed high-affinity binding sites with a dissociation constant of 130 pM with 1300 binding sites per cell while the low-affinity sites with a dissociation constant of 4.1 nM with 20,000 binding sites per cell (data not shown). VEGF_165_ also facilitated the motility of melanoma cells in both a chemotactic and chemokinetic manner, although it did not alter the proliferation of melanoma cells.

PlGF, which is about 40% homologous to VEGF-A at the amino acid level, binds specifically to VEGFR1 and induces various signaling pathways (Tammela *et al*. 2005; Shibuya 2006). In the current study, both PlGF and VEGF_165_ enhanced the migration of melanoma cells, suggesting that the VEGF_165_-induced migration of these cells is regulated via VEGFR1.

VEGFR1 and VEGFR2 belong to the receptor tyrosine kinase (RTK) subfamily and are known to induce the activation of several intracellular signaling molecules, including PI3K, Akt, Erk1/2, and p38 mitogen-activated protein kinase (MAPK), when they bind VEGF-A (Zhang *et al*. 2010; Szabo *et al*. 2016; Roskoski 2017). To determine whether the motility of melanoma cells is regulated by VEGFR1 or VEGFR2, the effects of inhibiting the tyrosine kinase activity of VEGFR1 or VEGFR2 on VEGF_165_-induced cell motility were investigated. TKIs of both VEGFR1 and VEGFR2 suppressed the migration of melanoma cells induced by VEGF_165_, although VEGFR2 TKI did not affect VEGF_165_-induced cell migration. The neutralizing antibody against VEGFR1 also suppressed VEGF_165_-induced cell migration. Furthermore, the transfection of an ASO targeting VEGFR1 markedly reduced the migration of melanoma cells, and the addition of VEGF_165_ did not increase the migration of melanoma cells transfected with this VEGFR1-targeting ASO. However, the migration of melanoma cells transfected with a VEGFR2-targeting ASO was not suppressed. Additionally, VEGF_165_ enhanced the migration of melanoma cells transfected with this VEGFR2-targeting ASO. Collectively, these findings suggest that the VEGF_165_-induced migration of melanoma cells is mediated though signaling involving VEGFR1.

The MAPK pathway is a canonical signaling pathway triggered by several RTKs (McKay and Morrison 2007; Tarcic and Yarden 2010) including VEGF receptors (Yu and Sato 1999). Therefore, we investigated whether VEGF_165_ induces the activation of the MAPK cascade in melanoma cells. We found that Erk is constitutively phosphorylated in SK-MEL-28 cells and VEGF_165_ did not affect the phosphorylation of Erk, indicating that VEGF_165_ is not involved in the MAPK pathway of melanoma cells. In addition to the MAPK cascade, the PI3K/Akt pathway is activated through RTKs (Matsuoka and Yashiro 2014; Mayer and Arteaga 2016; Nozhat and Hedayati 2016) including VEGF receptors (Yu and Sato 1999). In the melanoma cells tested in the present study, Akt was phosphorylated by VEGF_165_. To clarify whether VEGF_165_-induced Akt phosphorylation is regulated via VEGFR1 or VEGFR2, we investigated the effects of VEGFR1 and VEGFR2 TKIs on VEGF_165_-induced Akt phosphorylation. VEGFR1/2 TKIs suppressed the induction of Akt phosphorylation by VEGF_165_ in SK-MEL-28 cells, but the VEGFR2 TKI did not affect VEGF_165_-induced Akt phosphorylation. These findings show that VEGF_165_ induces phosphorylation of Akt via VEGFR1 in melanoma cells. We also examined the participation of PI3K in the VEGF_165_-induced Akt phosphorylation of melanoma cells, finding that the PI3K inhibitor wortmannin suppressed VEGF_165_-induced Akt phosphorylation in melanoma cells. Wortmannin also suppressed the VEGF_165_-induced migration of melanoma cells. These findings indicate that VEGF_165_ promotes the migration of melanoma cells through the activation of PI3K/Akt signaling via VEGFR1. Using recombinant human VEGFR1 shows that PI3-kinase binds directly to phosphorylated tyrosine residue 1213, which resulted from an autophosphorylation event (Yu *et al*. 2001).

In conclusion, the melanoma cells examined in this study produced VEGF_165_ and expressed RNAs encoding its receptors VEGFR1 and VEGFR2. However, these melanoma cell lines expressed VEGFR1 protein but not VEGFR2 protein. We found that VEGF_165_ enhanced cell motility via VEGFR1 but not VEGFR2. Thus, the motility of melanoma cells may be regulated by a VEGF_165_/VEGFR1-mediated autocrine signaling pathway. Moreover, we found that VEGF_165_-induced melanoma cell motility is mediated by the PI3K/Akt pathway via VEGFR1. A survey of 167 melanoma specimens found that less than 10% of the tumors expressed VEGFR2, and they suggested that anti-VEGF proliferation therapy would not be an effective strategy for melanomas (Molhoek *et al*. 2011). Our results suggest that VEGF-A/VEGFR1 signaling could serve as a therapeutic target to prevent the invasion and metastasis of melanoma with inhibition of the associated signaling pathway being a therapeutic strategy to treat melanoma.

## Supplementary Information

Below is the link to the electronic supplementary material.

Supplementary file1 Supplementary Figure S1 Confirmation of Mycoplasma-free culture. It was confirmed that all cell lines were free of mycoplasma contamination by PCR method. (PDF 291 KB)

Supplementary file2 Supplementary Table S1 STR profile of each cell line (PDF 240 KB)

## Data Availability

The data presented in this study are available on request from the corresponding author. Publicly available datasets were analyzed in this study.
