# Supplementary material for: VEGF-A promotes the motility of human melanoma cells through the VEGFR1–PI3K/Akt signaling pathway
Source: In Vitro Cell Dev Biol Anim. 2022 Aug 23;58(8):758–70. doi: 10.1007/s11626-022-00717-3 (PMC9550759; doi:10.1007/s11626-022-00717-3)
Supplement: Supplementary file 1 — Supplementary file1 Supplementary Figure S1 Confirmation of Mycoplasma-free culture. It was confirmed that all cell lines were free of mycoplasma contamination by PCR method. (PDF 291 KB) [file 11626_2022_717_MOESM1_ESM.pdf]

Figure S1. Koizumi K. et al.

Confirmation of Mycoplasma-free culture

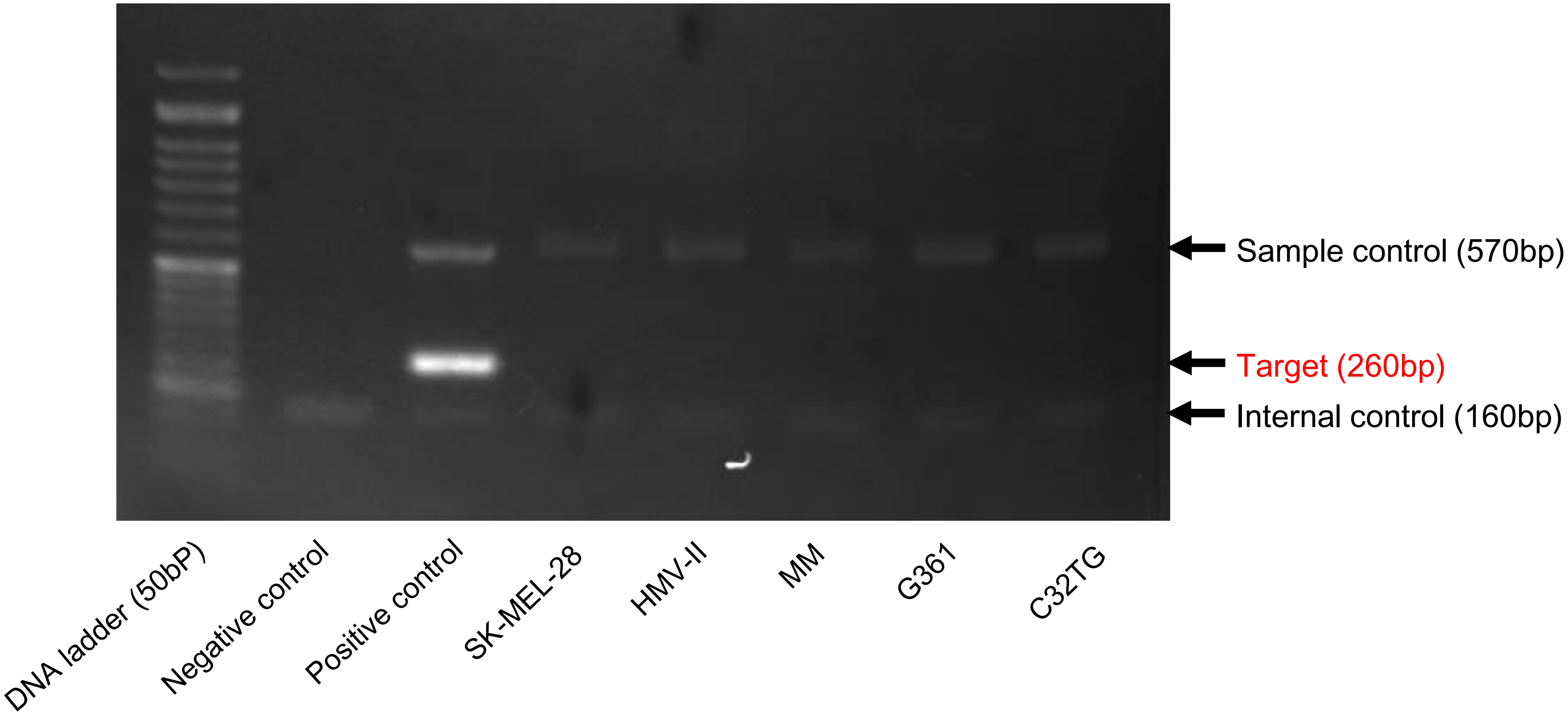

Kit; E-Myco plus Mycoplasma PCR Detection Kit (LiliF Diagnostics)

Genome DNA; 20ng

PCR; 35 cycles

1.2% agarose gel

20220701
