# Supplementary material for: VEGF-A promotes the motility of human melanoma cells through the VEGFR1–PI3K/Akt signaling pathway
Source: In Vitro Cell Dev Biol Anim. 2022 Aug 23;58(8):758–70. doi: 10.1007/s11626-022-00717-3 (PMC9550759; doi:10.1007/s11626-022-00717-3)
Supplement: Supplementary file 2 — Supplementary file2 Supplementary Table S1 STR profile of each cell line (PDF 240 KB) [file 11626_2022_717_MOESM2_ESM.pdf]

Supplementary Table 1. STR profile of each cell line

Locus	SK-MEL-28		HMV-Ⅱ		MM		G361			C32TG	
D3S1358	16	18	14	15	15		11	16		14	17
TH01	7		6	9	9		7	9		9.3	
D21S11	28	29	29		29	31	29	30		29	34.2
D18S51	12	16	15		12	13	15	16		12	15
Penta_E	8	12	5	21	15	23	10	14		12	
D5S818	12	13	12		10	12	11			11	12
D13S317	11	12	11		11	13	11			12	
D7S820	9.3	10	8	14	11		8	11		9	11
D16S539	9	12	11		10		15			11	12
CSF1PO	10	12	13		12	13	10			11	12
Penta_D	9	10	11		9	11	9	10	11	9	13
AMEL	X	Y	X		X		X			X	Y
vWA	18	19	16	17	14	18	15	17		14	19
D8S1179	12	13	10		13		12	13		13	14
TPOX	8	12	8		11	12	8	10		9	
FGA	19		23		19		19	21		23	24
